# Laughter therapy: A humor-induced hormonal intervention to reduce stress and anxiety

**DOI:** 10.1016/j.crphys.2021.04.002

**Published:** 2021-04-30

**Authors:** Nuraly S. Akimbekov, Mohammed S. Razzaque

**Affiliations:** a Department of Biotechnology, Al-Farabi Kazakh National University, Almaty, Kazakhstan; b Department of Pathology, Lake Erie College of Osteopathic Medicine, Erie, PA, USA

**Keywords:** Laughter, Anxiety, Insomnia, Depression

## Abstract

Prolonged pharmacological interventions have detrimental health consequences by developing drug tolerance or drug resistance, in addition to adverse drug events. The ongoing COVID-19 pandemic-related stress has adversely affected the emotional and mental health aspects around the globe. Consequently, depression is growing during the COVID-19 pandemic. Besides specific pharmacological interventions, which if prolonged have detrimental health consequences, non-pharmacological interventions are needed to minimize the emotional burden related to the COVID-19 pandemic. Laughter therapy is a universal non-pharmacologic approach to reduce stress and anxiety. Therapeutic laughter is a non-invasive, cost-effective, and easily implementable intervention that can be used during this pandemic as a useful supplementary therapy to reduce the mental health burden. Laughter therapy can physiologically lessen the pro-stress factors and increase the mood-elevating anti-stress factors to reduce anxiety and depression. In this ongoing stressful period of the COVID-19 pandemic, keeping necessary social distancing, it is important to create a cheerful environment that will facilitate laughter among the family, neighbor, and community to cope with the stresses of the COVID-19 pandemic.

## Highlights

• Prolonged pharmacological interventions have detrimental health consequences.
• Laughter therapy is a universal non-pharmacologic approach to reduce stress and anxiety.
• Therapeutic laughter is a non-invasive, cost-effective, and easily implementable intervention.
• Laughter therapy can be used during COVID-19 pandemic as a useful supplementary therapy to reduce the mental health burden.

## Graphical abstract

**Figure:**
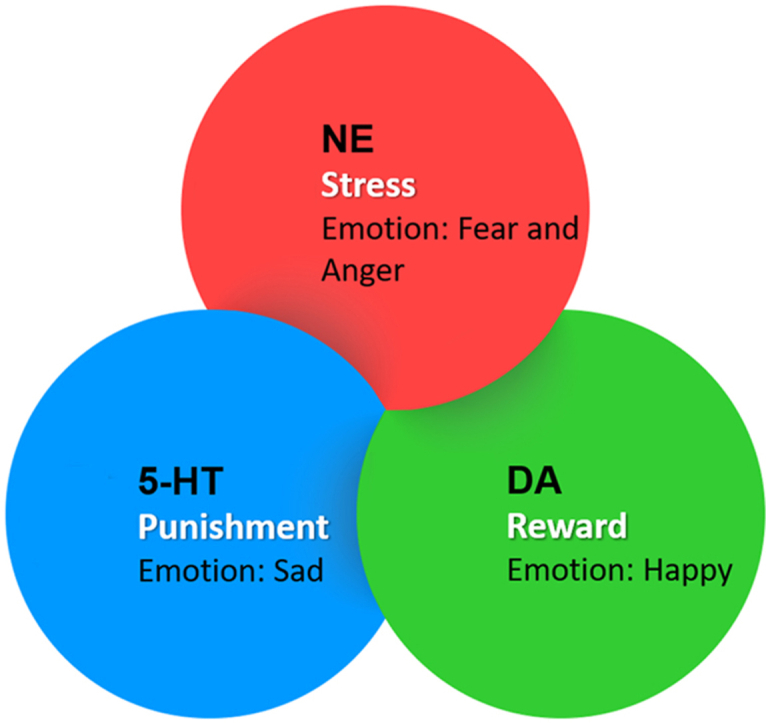

## 1 COVID-19 and stress

The ongoing COVID-19 pandemic-associated stress has impacted the quality of life of many people by negatively affecting the emotional and mental health aspects. Studies have shown that the number of individuals affected by depression has risen during the COVID-19 pandemic (Bareeqa et al., 2020; Bueno-Notivol et al., 2021). A population-based survey (U.S. adults aged 18 or older) has shown a three-fold increase in depression in the earlier months of the COVID-19 pandemic (Ettman et al., 2020). Additional surveys (conducted by multiple U.S. academic institutes from Indiana, New Mexico, Florida, and Texas) have found more than one-third of the adult U.S. population are having COVID-19-related distress (Khubchandani et al., 2021); the prevalence of and psychological distress (39%), anxiety (42%), and depression (39%) were also estimated by analyzing 1978 individuals across the U.S. (Khubchandani et al., 2021). Such COVID-19 pandemic-related stress, anxiety, and depression are likely to progress into debilitating mental illness, if timely intervention is not offered. Of relevance, persistent stressors can impair mental health and induce cardiovascular complications and metabolic diseases, including obesity and diabetes (Arigo et al., 2020; Cuevas et al., 2020; Gomez-Perez et al., 2020; Miller et al., 2020; Walker et al., 2020). The COVID-19-related fear of health risks, amalgamated with the anxiety of economic hardship, is preferentially affecting the vulnerable populations, including women, the elderly, and low resource communities (Ettman et al., 2020). Overuse and misuse of drugs in the management of COVID-19-associated signs and symptoms are a serious health concern, and finding non-pharmacological interventions to reduce pandemic-associated health hazards is an urgent necessity. Of importance, exacerbation of antimicrobial resistance due to unrestricted use of antimicrobial drugs appears to be another casualty of the COVID-19 pandemic (Razzaque, 2020e). Implementing antimicrobial stewardship to minimize the needless use of antimicrobial therapies to lessen the drug resistance in this pandemic should be a medical priority (Razzaque, 2020b; f).

## 2 Laughter as an intervention

**Fig. 1:**
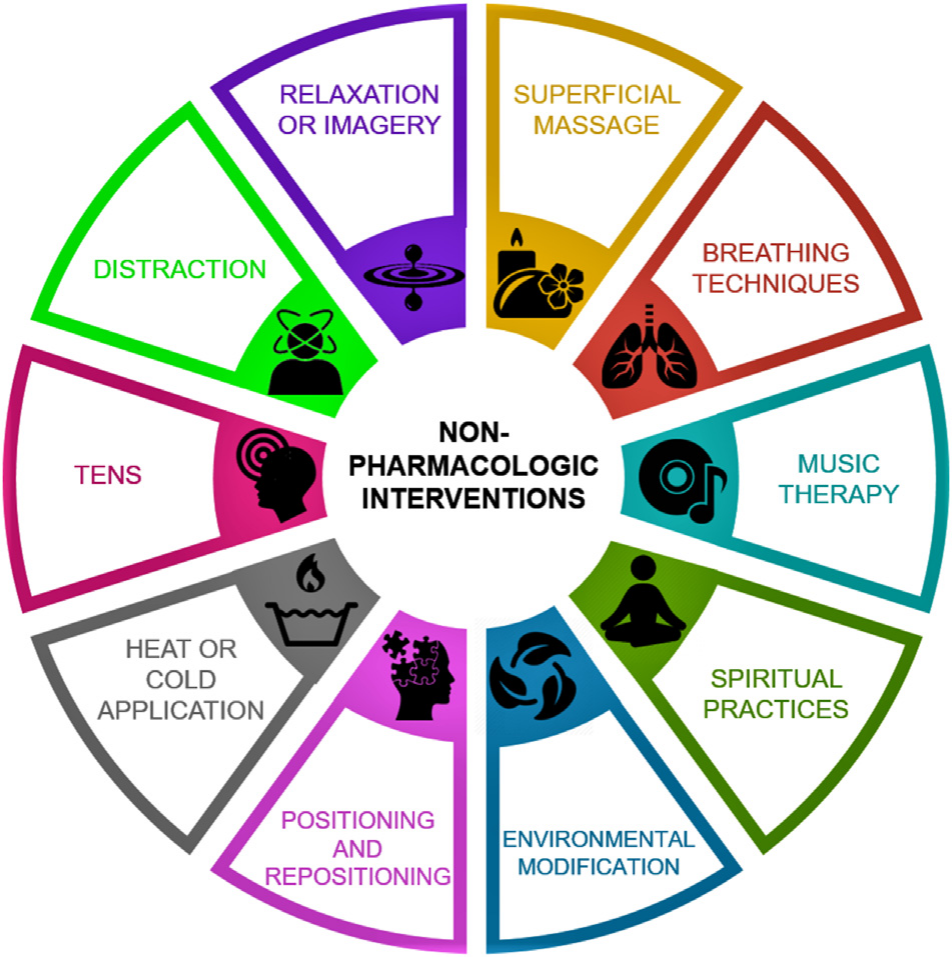
Major types of non-pharmacologic interventions.

Non-pharmacologic interventions are recognized as being a useful approach to reduce pain, stress, and anxiety. Nonpharmacologic interventions range from superficial massage to breathing exercise to music therapy to yoga to spiritual practices (Lewis et al., 2018) (Fig. 1). Laughter therapy, one of the key non-pharmacological interventions, is a universal approach to reduce stress and anxiety. From ancient times, laughter has been used to influence cognitive behavior to improve and establish healthy physical, psychological, and social relationships. Studies have documented the positive role of laughter in enhancing the quality of life (Kuru and Kublay, 2017; Heidari et al., 2020). Laughter therapy may be used for both preventive and therapeutic purposes. Laughter can also be a clinical predictor of functional disability. An analysis of 14,233 elderly individuals (aged ≥65 years), selected for the ‘Japan Gerontological Evaluation Study’, a low frequency of laughter has shown to be linked to a higher risk of developing functional disability (Tamada et al., 2020); adjusting the potential confounders, the investigators reported an increased hazard ratio of functional disability among the individuals with decreased frequency of laughter. About 1.42 times higher risk of functional disability was noted among the individuals with low or no laughing habits (Tamada et al., 2020).

There is a biological explanation of how laughter is reducing stress, anxiety, and depression. Laughter has been shown to exert stress-reducing effects by suppressing the bioactivities of epinephrine, cortisol, and 3,4-dihydrophenylacetic acid (a major dopamine catabolite) (Berk et al., 1989; Yim, 2016). Reduced neurotransmitter activities, including norepinephrine, serotonin, and dopamine are linked to depression, and laughter is shown to enhance dopamine and serotonin activities (Yim, 2016). Emotion is an expression from the mixture of the three monoamine neurotransmitters: norepinephrine, dopamine, and serotonin (Wang et al., 2020). A three-dimensional model for monoamine neurotransmitters and basic emotions is suggested, where each basic emotion has its own concentration level of neurotransmitters (Lovheim, 2012). The model was based on an elaborate and comprehensive theory of basic emotions, where human emotions were divided into one neutral (surprise/startle), two positive (interest/excitement and enjoyment/joy), and five negatives (distress/anguish, fear/terror, shame/humiliation, contempt/disgust, and anger/rage) categories, and further connects them with facial expressions, and posture as well as typical physiological manifestations (Tomkins and Mccarter, 1964; Tomkins, 1975; Atwood and Tomkins, 1976). Gu et al. proposed a “three primary color model” of basic emotions: norepinephrine is responsible for fear and anger emotions, joy is subsided by dopamine, while punishment is subsided by serotonin (Gu et al., 2018). Many follow-up studies have supported this emotion theory and reported that three monoamines (as substrates) might play a central role in emotional expression, including laughter (Gu et al., 2016, 2018; Wang et al., 2020).

A randomized controlled trial of laughter intervention on patients with schizophrenia has found differential regulation of brain-derived neurotrophic factor (BNDF) and cortisol, when significantly higher serum levels of BDNF were recorded following 8-week of simulated laughter therapy, the level of BNDF dropped following discontinuation of laughter intervention (Cheng et al., 2020). No change in cortisol level was noted following 8 weeks of laughter therapy (Cheng et al., 2020). Whether simulated laughter intervention can be used as an alternative or complementary treatment or as a social support system for rehabilitation of patients with schizophrenia to reduce their stress responses will need additional studies, but the potential exists for such non-pharmacology intervention as a supportive treatment (Cheng et al., 2020).

Laughter therapy has been shown to benefit a wide range of non-COVID-19 patients. A Japanese study conducted on 41 female patients with rheumatoid arthritis has shown that the basal level of serum growth hormone in rheumatoid arthritis patients was markedly elevated, as compared to the healthy control subjects, and the level of growth hormone significantly reduced in rheumatoid arthritis patients following laughter therapy (Ishigami et al., 2005). In a similar line of study, the basal levels of serum proinflammatory cytokines, Interleukin 6 (IL-6) and Tumor Necrosis Factor alpha (TNF-alpha), were markedly higher in the rheumatoid arthritis patients than the healthy controls; the serum levels of IL-6 and TNF-alpha significantly reduced in the rheumatoid arthritis patients after laughter intervention (Matsuzaki et al., 2006). Additionally, the laughter-induced release of endorphins can help in reducing depressed mood (Lebowitz et al., 2011). Without even pharmacological intervention, laughter therapy can physiologically lessen the pro-stress factors and increase the mood-elevating anti-stress factors to reduce the stress responses, including anxiety and depression (Bennett and Lengacher, 2009). Laughter can also assist muscle relaxation and increase circulation to reduce the physical symptoms of stress. Moreover, laughter may decrease pain sensation by facilitating the synthesis of natural painkillers. In addition, studies have shown that laughter can enhance the tolerance of pain (Dunbar et al., 2012; Lapierre et al., 2019).

Although laughter is the natural expression of positive emotion, it can be broadly grouped into five different categories (Table 1). Laughter has a wide range of benefits, ranging from increase cognitive functions to improve respiration to enhance pain tolerance threshold to reduce stress hormones, with cumulative effects being the improved psychological well-being (Scholl and Ragan, 2003; Sahakian and Frishman, 2007; Yim, 2016). Hence, laughter therapy is used to augment psychosocial behaviors to improve the overall quality of life (Ko and Youn, 2011). Depression is one of the frequently observed mental disorders, and clinical depression needs pharmacological intervention. Laughter can mitigate the adverse consequences of stress and reduce depression by releasing neurotransmitters (Farifteh et al., 2014). While it is not always possible to dissociate confounding factors, laughter therapy tends to increase the sense of well-being across age groups and gender, including vulnerable elderly individuals (Gonot-Schoupinsky and Garip, 2018). In a meta-analysis on 814 participants from 10 published studies, laughter interventions were reported to significantly reduce depression and anxiety levels, along with an increase in better quality of sleep; the benefits on depression were more pronounced with long-term laughter intervention (Zhao et al., 2019). In a randomized controlled trial (aged ≥60 years), a laughter therapy program lead to reduced anxiety and insomnia among the elderly participants, with the resultant effect being the overall improvement of the general health (Ghodsbin et al., 2015). Similar positive effects on depression and insomnia were also documented following laughter therapy among the community-dwelling (≥60 years of age and living independently) elderly individuals in South Korea (Ko and Youn, 2011). In another study on 26,368 elderly Japanese individuals (men, 12,174; women, 14,194; aged ≥60 years), after adjusting for depression, sociodemographic factors, and social participation, the prevalence ratio for poor subjective health was noted among women who hardly laughed as compared with those who reported to have daily laughing habits (Hayashi et al., 2015). Of relevance, laughter in the elderly population is associated with the status of their oral health. In another Japanese study, conducted on the 11,239 male and 12,799 female community-dwelling elderly individuals, after adjusting for all covariates, the participants with 10 or more teeth were more likely to laugh than the edentulous (lacking teeth) participants (Hirosaki et al., 2021). The association between (the status of) oral health and (subsequent implications on) general health is well-documented in various systemic diseases, including diabetes and cardiovascular diseases.

**Table 1:** Five main categories of laughter (Yim, 2016).

• Spontaneous laughter (triggered by positive emotion: unrelated to free will)
• Simulated laughter (triggered by oneself at will: self-induced)
• Stimulated laughter (triggered by physical contact: ticklish)
• Induced laughter (triggered by drug: nitrous oxide)
• Pathological laughter (triggered by neuronal damage: pseudobulbar affect)

## 3 Conclusion and recommendation

**Fig. 2:**
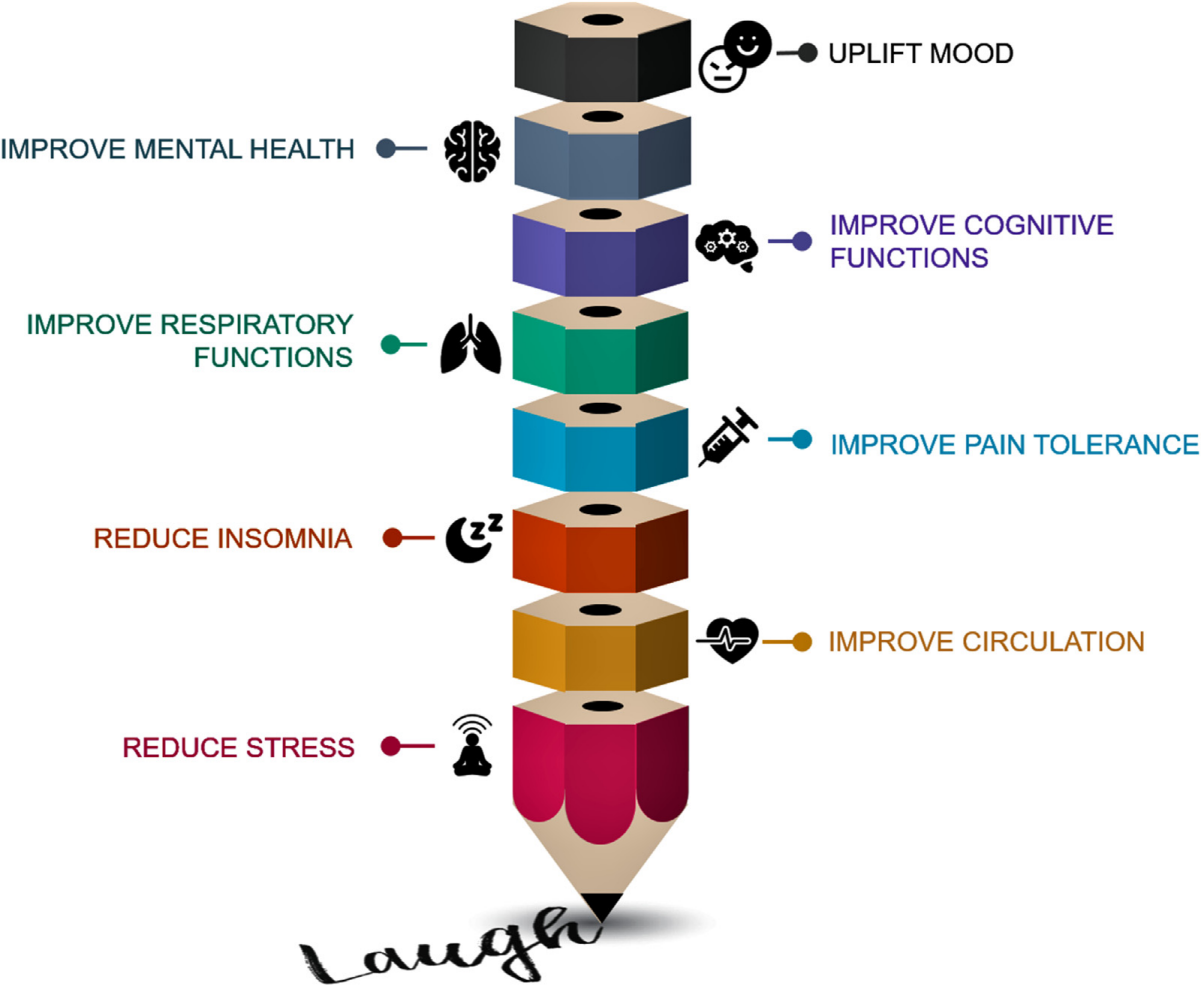
A few potential benefits of laughter therapy.

Laughter is a human asset, and effectively using laughter to minimize short-term and long-term stresses can significantly improve the quality of life (Zhao et al., 2019). The potential benefits of laughter are summarized in Fig. 2. In fact, in this ongoing stressful period of COVID-19 pandemic, creating a cheerful environment to laugh is perhaps more important than ever to cope with the stresses related to the COVID-19 pandemic. Therapeutic laughter is a non-invasive, cost-effective and easily implementable intervention that can be used as an effective complementary therapy to reduce the intensity of many mental illnesses (Van Der Wal and Kok, 2019). However, the effects of laughter on the neuroendocrine, and immune systems are needed to be objectively studied to define the underlying molecular regulation of psychosocial behaviors.

In the absence of specific pharmacological therapy for COVID-19, non-pharmacological interventions (Fig. 1) to lessen emotional and physical stresses related to COVID-19 are essential. Such non-pharmacological interventions and community compliance are required to attain the desired goals of minimizing COVID-19 transmission and reduce stress-related burdens. It appears likely that maintaining an adequate nutritional balance through healthy eating habits, keeping an active lifestyle, along with practicing laughter can help in reducing the stress-related disease burden of COVID-19 pandemic and other chronic debilitating diseases (Razzaque, 2020d; c;a; Abukabda and Razzaque, 2021; Razzaque, 2021).

## Author contributions

Mohammed S. Razzaque conceptualized, designed, drafted and edited the manuscript. Nuraly S. Akimbekov edited the manuscript and contributed to the artworks.

## Declaration of competing interest

The authors declare that they have no known competing financial interests or personal relationships that could have appeared to influence the work reported in this paper.
